# Catatonia associated with late-life psychosis successfully treated with lithium: a case report

**DOI:** 10.1186/s12991-021-00336-4

**Published:** 2021-02-18

**Authors:** Hiroko Sugawara, Junpei Takamatsu, Mamoru Hashimoto, Manabu Ikeda

**Affiliations:** 1 Department of Psychiatry, Kansai Rosai Hospital, 3-1-69 Inabasou, Amagasaki, Hyogo 660-8511 Japan; 2 Department of Psychiatry, Osaka University Graduate School of Medicine, Osaka, Japan; 3 Department of Emergency Medicine, Kansai Rosai Hospital, Amagasaki, Japan

**Keywords:** Catatonia, Late-life psychosis, Lithium, Benzodiazepine, Psychotic symptoms

## Abstract

**Background:**

Catatonia is a psychomotor syndrome that presents various symptoms ranging from stupor to agitation, with prominent disturbances of volition. Its pathogenesis is poorly understood. Benzodiazepines and electroconvulsive therapy (ECT) are safe and effective standard treatments for catatonia; however, alternative treatment strategies have not been established in cases where these treatments are either ineffective or unavailable. Here, we report a case of catatonia associated with late-life psychosis, which was successfully treated with lithium.

**Case presentation:**

A 66-year-old single man with hearing impairment developed hallucination and delusions and presented with catatonic stupor after a fall. He initially responded to benzodiazepine therapy; however, his psychotic symptoms became clinically evident and benzodiazepine provided limited efficacy. Blonanserin was ineffective, and ECT was unavailable. His catatonic and psychotic symptoms were finally relieved by lithium monotherapy.

**Conclusions:**

Catatonic symptoms are common in patients with mood disorders, suggesting that lithium may be effective in these cases. Moreover, lithium may be effective for both catatonic and psychotic symptoms, as it normalizes imbalances of excitatory and inhibitory systems in the brain, which underlies major psychosis. Cumulative evidence from further cases is needed to validate our findings.

## Background

Catatonia is a psychomotor syndrome that presents various symptoms ranging from stupor to agitation, with prominent disturbances of volition; its pathogenesis is poorly understood [1]. Catatonia was once recognized as a subtype of schizophrenia; however, the Diagnostic and Statistical Manual of Mental Disorders fifth edition (DSM-5) removed it from all schizophrenia subtypes, and defined it as a specifier of various psychiatric disorders or medical conditions [2]. Indeed, catatonia has been reported to be associated with a variety of medical conditions [3, 4]. Moreover, a previous study has reported that catatonic symptoms were more common in patients with manic or mixed episodes (28–31%) than in those with schizophrenia (10–15%) [5]. A prospective cohort study has also reported the incidence of catatonia to be only 7.6% in patients with schizophrenia [6].

Benzodiazepines and electroconvulsive therapy (ECT) are safe and effective standard treatments for catatonia, particularly in acute cases; however, benzodiazepines show limited efficacy in a considerable number of patients [7], and available facilities for ECT are limited. In cases where benzodiazepines are ineffective and ECT is unavailable, a systematic review of alternative treatment strategies proposed glutamate antagonists, antiepileptic drugs, and atypical antipsychotics as first-, second-, and third-line treatments for catatonia, respectively [8]. The proper alternative treatment of catatonia may differ depending on the underlying disease; however, as its pathogenesis is unclear, the pharmacological action of each drug is therefore unknown.

Here, we report a case of catatonia associated with late-life psychosis that was successfully treated with lithium.

## Case presentation

**Fig. 1:**
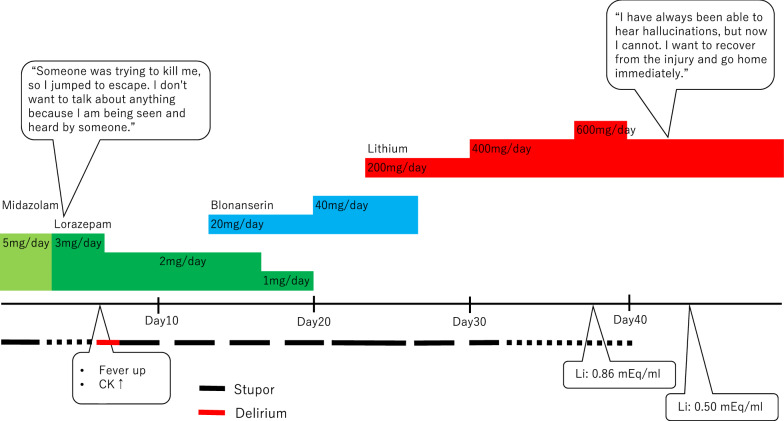
Clinical course of a case of catatonia associated with late paraphrenia. A 66-year-old man with catatonic stupor initially responded to benzodiazepine therapy, and his psychotic symptoms became clinically evident because he stated that “someone was trying to kill me, so I jumped to escape. I don’t want to talk about anything because I am being seen and heard by someone.” He subsequently developed delirium with fever and creatine kinase (CK) level elevation and the dose of lorazepam was tapered to 2 mg/day. After normalization of CK levels, a blonanserin patch at a dose of 20 mg/day was added for his underlying psychotic symptoms. The dose of the blonanserin patch was increased to 40 mg/day, and lorazepam was terminated; however, it was not effective. Although he was a suitable candidate for ECT, it was unavailable in our facility. Lithium was, therefore, added, and the blonanserin patch was terminated; his catatonic and psychotic symptoms were finally relieved by lithium monotherapy. He said that “I have always been able to hear hallucinations, but now I cannot. I want to recover from the injury and go home immediately.” Although the dose of lithium was temporarily increased to 600 mg/day (0.86 mEq/ml), the dose of 400 mg/day (0.50 mEq/ml) was maintained after obtaining informed consent, based on the blood concentration results. *Li* blood concentration of lithium, *CK* creatine kinase

The patient was a 66-year-old single man, who worked for a cleaning company, and had hearing impairment; however, there was no evident medical history of psychiatric disorders. He was found lying in front of his apartment and was sent to the emergency room. A physical examination revealed fracture of the left patella and calcaneus, which appeared to be related to trauma. He kept his eyes closed for most of the day and demonstrated no spontaneous speech or reaction. Although his blood tests showed inflammatory reaction, his vital signs were normal, and computed tomography and electroencephalography of his brain showed no significant findings. He had no relatives and there was no life history information of him. According to the information of the neighboring residents, he had exhibited strange behaviors since about 2 months before admission and often annoyed the neighbors. Before his arrival to the emergency department, a neighbor called the police because the patient had stood in front of his apartment for a long time. While in detention, he did not respond to the police interrogations at all; he was found lying in front of his apartment a few hours after police released him. After admission to the emergency department, he was consulted with our department for the assessment of psychotic symptoms by his physician. Including the process leading to hospitalization, he was diagnosed with catatonia based on the presence of stupor, mutism, and negativism which was detected from the information by the police about the patients’ uncooperative behavior during detention. He was started on intravenous administration of 5 mg/day midazolam, which was switched to lorazepam, administered through a feeding tube. He then gradually opened his eyes and started speaking. He stated that “someone was trying to kill me, so I jumped to escape. I don’t want to talk about anything because I am being seen and heard by someone.” He was, therefore, suspected to have hallucinations and delusions. He subsequently developed fever, and the blood tests showed an inflammatory reaction with creatine kinase (CK) elevation; antibiotics were accordingly administered for infection at the injury site. The dose of lorazepam was tapered to 2 mg/day because he developed delirium; after normalization of CK levels, a blonanserin patch at a dose of 20 mg/day was added for his underlying psychotic symptoms. His delirium improved, and he was able to eat by himself; however, he spent more time lying down with his eyes closed. Considering the possibility of oversedation, lorazepam was tapered to a dose of 1 mg/day. Since he appeared to have improved slightly, the dose of blonanserin patch was increased to 40 mg/day, and lorazepam was terminated. However, he spent most of his time in bed, and eating became difficult. Although he was a suitable candidate for ECT, it was unavailable in our facility. We tried to transfer the facility where ECT was available; however, it was full. Lithium was, therefore, added, and blonanserin patch was terminated; he opened his eyes and began to move after the dose of lithium was increased to 400 mg/day, and he began to eat and talk. He said that “I have always been able to hear hallucinations, but now I cannot. I want to recover from the injury and go home immediately.” Although the dose of lithium was temporarily increased to 600 mg/day, the dose of 400 mg/day was maintained after obtaining informed consent, based on the blood concentration results (Fig. 1). He mentioned that he had graduated from high school and had no history of alcohol or drug abuse. At age 60, he moved to his present apartment after changing jobs, and had developed hallucinations approximately a year before hospitalization. On examination, his mini-mental state examination (MMSE) score was 26; some points were lost on 3-step command (minus 2) and delayed recall (minus 2) tasks, suggesting that there was no remarkable cognitive dysfunction. He was, therefore, transferred to a rehabilitation facility.

## Discussion and conclusions

This report describes the case of a single elderly man with hearing impairment, who developed hallucination and delusions and presented with catatonia at the last minute before his fall. There were no remarkable brain organic abnormalities which possibly cause catatonic symptoms, suggesting that his psychotic and catatonic symptoms were not derived from either trauma or drugs used after admission. His premorbid social function was almost normal enough to work for cleaning company, and the results of cognitive function tests performed after his psychiatric symptoms improved showed that he did not have remarkable neurocognitive impairment leading to social dysfunction. He had sensory deficit, which is common in late paraphrenia [9], and developed psychotic symptoms after 60 years of age without any evident history of psychiatric disorders. This suggested that his catatonia occurred because of late-life psychosis called as late paraphrenia, which has been recognized to be independent of schizophrenia [10]. Although the patient showed no remarkable cognitive dysfunction in MMSE, we could not rule out the possibility of neurocognitive deficit of the patient because there was no other neurocognitive examination of the patient to assess cognitive impairment. A previous review of paraphrenia has reported that the pathology of paraphrenia is similar to that of the neurofibrillary tangles, which are the predominant form of senile dementia [11]. His psychotic episode could have represented the prodromal symptoms of dementia; he therefore needs to be followed up closely for the progressive of cognitive dysfunction in the future.

The patient had initially responded to benzodiazepine therapy, and his psychotic symptoms became clinically evident; however, its efficacy was limited. Some patients with catatonia fail to respond to benzodiazepines, with approximately 30% of patients showing only partial response [12, 13]; in particular, cases that occur secondary to schizophrenia have been reported to be less likely to respond to benzodiazepines [14, 15]. Moreover, benzodiazepine use is not suitable for elderly patients, considering the risk of delirium. Atypical antipsychotics have been recommended as one of the alternative treatment strategies for catatonia [8]; however, blonanserin did not improve his catatonia. Finally, both his catatonic and psychotic symptoms were completely resolved by lithium monotherapy. Catatonic symptoms are more common in patients with mood disorders than in those with schizophrenia [5], suggesting that lithium may be effective for catatonia. Although previous case reports advocate the effectiveness of lithium in preventing recurrence of catatonia [16, 17], there is no evidence of its effectiveness in the acute phase.

Although the pathogenesis of catatonia remains poorly understood, the neurochemical hypothesis suggests that alterations in various neurotransmission systems, including gamma-aminobutyric acid (GABA) and glutamate, play a role [18, 19]. Even if the effects of benzodiazepines on the GABA system in the brain are limited, ECT has a high response rate, suggesting that catatonia may be the final common outcome for abnormal brain seizure activity [20]. In the present case, antiepileptic drugs which are candidates of the alternative treatment strategies for catatonia [8] were not administrated due to no remarkable abnormalities of electroencephalography. Finally, lithium was used because the effect of benzodiazepine was limited and ECT was unavailable. A previous study, using induced pluripotent stem cells (iPSCs) derived from neuronal cells of patients with bipolar disorders, has reported that the hyperexcitability phenotype of young neurons was selectively reversed by lithium only in lithium responders [21]. Moreover, a recent study using iPSCs derived from monozygotic twins discordant for major psychosis has suggested that lithium may normalize unbalanced specification of excitatory and inhibitory neurons in major psychosis neural circuits, by activating the Wnt signaling pathway [22]. In the present case of catatonia associated with late-life psychosis, lithium, although not as fast-acting as ECT, was effective for both catatonic and psychotic symptoms, as it normalized unbalanced specification of excitatory and inhibitory systems in the brain.

To the best of our knowledge, this is the first report on the efficacy of lithium in the acute phase of catatonia. There was a previous report of lithium therapy for catatonia features in autism spectrum disorder patients [23] and the another reported the case of catatonia cause by lithium overdose [24], suggesting that the action and effects of lithium on neurons numerous and diverse. Future studies are needed to elucidate the pathogenesis of catatonia to identify the most reliable treatment.

## Data Availability

The data used for this case report is available from the corresponding authors on reasonable request.

## Abbreviations

ECT: Electroconvulsive therapy
iPSCs: Induced pluripotent stem cells
